# Temporal elevation of blood pressure is associated with increased risk of sudden cardiac arrest

**DOI:** 10.1038/s41598-024-52859-x

**Published:** 2024-01-27

**Authors:** Yun Gi Kim, Kyongjin Min, Joo Hee Jeong, Seung-Young Roh, Kyung-Do Han, Jaemin Shim, Jong-Il Choi, Young-Hoon Kim

**Affiliations:** 1 Division of Cardiology, Department of Internal Medicine, Korea University College of Medicine and Korea University Anam Hospital, 73 Goryeodae-Ro, Seongbuk-Gu, Seoul, 02841 Republic of Korea; 2 Division of Cardiology, Department of Internal Medicine, Incheon Sejong Hospital, Incheon, Republic of Korea; 3 Division of Cardiology, Department of Internal Medicine, Korea University College of Medicine and Korea University Guro Hospital, Seoul, Republic of Korea; 4 Department of Statistics and Actuarial Science, Soongsil University, Seoul, Republic of Korea

**Keywords:** Cardiology, Risk factors

## Abstract

Hypertension is a known risk factor for sudden cardiac arrest (SCA). However, the role of temporal changes in blood pressure on the risk of SCA is not fully understood. This study was conducted to determine whether a temporal increase or decrease in blood pressure is associated with the risk of SCA. This study was based on nationwide healthcare insurance data. Individuals who underwent nationwide health check-ups in 2009 and 2011 were analyzed. A total of 2,801,153 individuals were evaluated for 8100 SCA events during the 17, 740, 420 person-years of follow-up. In a multivariate analysis, there were linear association between the degree of temporal elevation of systolic blood pressure (SBP) and the risk of SCA: (i) adjusted-hazard ratio (HR) 1.11 (p = 0.001) in 10 ≤ ΔSBP < 20 (mmHg) group; (ii) adjusted-HR 1.40 (p < 0.001) in 20 ≤ ΔSBP < 40 group; and (iii) adjusted-HR 1.88 (p < 0.001) in 40 ≤ ΔSBP group as compared with the reference group (− 10 ≤ ΔSBP < 10). Temporal increase in diastolic blood pressure (DBP) also a showed significant association with SCA risk with the highest risk observed in ∆DBP ≥ 25 group (adjusted-HR 1.61; p < 0.001) as compared with the reference group (− 5 ≤ ΔDBP < 5). The association between SBP and SCA was not affected by age, sex, presence of diabetes mellitus, or baseline SBP. In conclusion, a temporal increase in blood pressure was significantly associated with the occurrence of SCA, and this association was consistent across all subgroups. However, a temporary decrease in blood pressure does not reduce the risk of SCA. Prevention of elevated blood pressure may play an important role in preventing SCA.

## Introduction

Sudden cardiac arrest (SCA) is one of the most emergent medical condition which require immediate therapeutic intervention to bring the victim back to life^1–3^. Various efforts intended to improve neurologically intact survival rate in SCA patients were attempted^4–9^. Although widespread supply of automatized defibrillators, high quality education of the citizens, and early initiation of bystander cardiopulmonary resuscitation have been demonstrated to improve survival of SCA patients^7–9^, it is uncertain whether other therapeutic interventions such as induced hypothermia, antiarrhythmic drugs, and coronary angiography can be beneficial to SCA victims^4–6^. Therefore, primary prevention of SCA, rather than treatment, can have a more profound impact on public health, preserving medical resources.

Hypertension is a known risk factor for SCA^10^. Our previous study demonstrated that hypertension and prehypertension were both associated with a significantly increased risk of SCA, and the risk can be even higher if combined with diabetes mellitus^10^. However, it is unclear whether temporal changes in blood pressure are associated with the risk of SCA. Elevated blood pressure during a certain period can aggravate atherosclerosis of the cerebral and coronary arteries, which can lead to cerebrovascular accidents and acute coronary syndrome, thereby increasing the risk of SCA^11–13^. In contrast, a temporary decrease in blood pressure may stabilize atherosclerotic cardiovascular disease and decrease the risk of SCA.

We aimed to evaluate whether temporal elevation in blood pressure is associated with an increased risk of SCA using nationwide population-based data from the Republic of Korea. We further analyzed whether maintaining a similar blood pressure or decreasing blood pressure is better for the prevention of SCA, as this issue has not been fully addressed in prior studies.

## Material and methods

### Database

The Korean National Health Insurance Service (K-NHIS) database was used in this study. All people in the Republic of Korea are mandatory subscribers of the K-NHIS; therefore, the entire population of the Republic of Korea is represented by data derived from the K-NHIS. The K-NHIS is distinguished from other claims data-based medical cohorts because it offers a regular health checkup program to its subscribers. During the nationwide health check-up, various laboratory tests, medical measurements, and self-questionnaires were provided and stored in a database. Therefore, medical researchers can analyze (i) blood test results, such as complete blood cell counts, liver and renal function tests, lipid profiles, fasting blood glucose (FBG), and gamma-glutamyl transferase levels; (ii) measurements of systolic and diastolic blood pressure (SBP and DBP), body weight, height, and waist circumference; and (iii) lifestyle factors, including smoking status, alcohol consumption, and exercise level. Importantly, a nationwide health check-up program is provided to subscribers once every 2 years which enables the evaluation of temporal changes in various parameters, including blood pressure. The K-NHIS database also contains reports of the International Classification of Disease, tenth edition (ICD-10) diagnostic codes, and prescription histories of all government-approved drugs available in the Republic of Korea.

Medical researchers were permitted to use the K-NHIS database with approval from relevant institutional review boards and the official review committee of the K-NHIS (https://nhiss.nhis.or.kr/). This study was approved by the institutional review board of the Korea University Medicine Anam Hospital and the K-NHIS review committee. The requirement for written informed consent was waived by the Institutional Review Board of Korea University Medicine Anam Hospital owing to the retrospective nature of the study. The legal regulations of the Republic of Korea and the ethical guidelines of the 2013 Declaration of Helsinki were followed throughout this study.

### Definitions

In this study, ∆SBP and ∆DBP were defined as temporal changes in SBP and DBP during 2009 and 2011 nationwide health check-ups. Based on a self-questionnaire during the 2009 health check-up, smoking status was defined as follows: (i) current smokers: those who smoked at least 100 cigarettes in their lifetime and continued smoking within 1 month of the 2009 health check-up; (ii) ex-smokers: those who smoked at least 100 cigarettes in their lifetime but had not smoked within 1 month of the 2009 health check-up; and (iii) never smokers: those who smoked < 100 cigarettes in their lifetime. Participants were classified into three groups according to the total amount of alcohol consumed per week: (i) non-drinkers: 0 g per week, (ii) mild drinkers: less than 210 g but more than 0 g per week, and (iii) heavy drinkers: 210 g or more per week. Diabetes mellitus was diagnosed if an individual had a measured fasting blood glucose (FBG) ≥ 126 mg/dL during a 2009 health check-up or a prior history of physician-diagnosed diabetes. The diagnosis of hypertension was based on ICD-10 codes for hypertension, systolic blood pressure ≥ 140 mmHg, or diastolic blood pressure ≥ 90 mmHg during the 2009 health checkup. Dyslipidemia was defined based on a prior diagnosis of dyslipidemia by a physician. The estimated glomerular filtration rate was calculated based on creatinine levels measured during the 2009 health check-up. Heart failure was identified based on ICD-10 (I50 and its sub-codes) codes for heart failure reported during in-hospital admissions. Cardiovascular disease was defined as prior reports of ICD-10 codes for myocardial infarction (I21 and I22) or ischemic stroke (I63 and I64) at either inpatient or outpatient setting. Our previous studies demonstrated the robustness of these definitions^14–17^.

### Participants

All patients who underwent serial nationwide health check-ups between 2009 and 2011 were screened for eligibility. The exclusion criteria were (i) prior diagnosis of SCA prior to the 2009 health check-up, (ii) age < 20 years, and (iii) SCA or death that occurred within 1 year after the 2011 health screening. Data obtained from January 2002 to December 2008 were used to identify baseline medical history such as hypertension, diabetes mellitus, heart failure, or dyslipidemia. Medical follow-up data until December 2018 were available.

Study participants were classified into six groups based on ∆SBP between 2009 and 2011: (i) ∆SBP < − 20 mmHg, (ii) − 20 mmHg ≤ ∆SBP < − 10 mmHg, (iii) − 10 mmHg ≤ ∆SBP < 10 mmHg, (iv) 10 mmHg ≤ ∆SBP < 20 mmHg, (v) 20 mmHg ≤ ∆SBP < 40 mmHg, and (vi) ∆SBP ≥ 40 mmHg. For ∆DBP, study participants were classified as follows: (i) ∆DBP < − 15 mmHg, (ii) − 15 mmHg ≤ ∆DBP < − 5 mmHg, (iii) − 5 mmHg ≤ ∆DBP < 5 mmHg, (iv) 5 mmHg ≤ ∆DBP < 15 mmHg, (v) 15 mmHg ≤ ∆DBP < 25 mmHg, and (vi) ∆DBP ≥ 25 mmHg.

### Primary outcome endpoint

The main outcome of this study was the occurrence of SCA which was identified through the reporting of ICD-10 codes during visits to the emergency department, excluding incidents in inpatient or outpatient settings: I46.0 (cardiac arrest with successful resuscitation), I46.1 (sudden cardiac arrest), I46.9 (cardiac arrest, cause unspecified), I49.0 (ventricular fibrillation and flutter), R96.0 (instantaneous death), and R96.1 (death occurring less than 24 h from symptom onset). If a prior diagnosis of sepsis (A40 or A41), cerebral hemorrhage (I60–I62), ischemic stroke (I63 or I64), asphyxia (R09.0), gastrointestinal bleeding (K25.0, K25.2, K25.4, K26.0, K26.2, K26.4, K26.6, K27.0, K27.2, K27.6, K28.0, K28.4, K28.6, K29.0, K92.0, K92.1, or K92.2), anaphylaxis (T78), trauma (S00–S99), or consequences of external causes such as poisoning, suffocation, drowning, hit by lightning, electric shock, or burn (T00–T98) was made within 6 months of the occurrence of SCA, that event was not counted as the primary outcome endpoint. To prevent errors arising from repeated reports of ICD-10 codes for SCA, reports of SCA or death that occurred within 1 year after the 2011 health screening were not counted as the main outcome. For example, a report of SCA codes immediately after health screening can be an actual SCA event after the 2011 health screening or just a repeated report of aborted SCA events that occurred before the 2011 health screening. Both aborted and non-aborted SCA were included as primary outcomes. The incidence of SCA was defined as the numbers per 1000 person-years of follow-up.

### Statistical analysis

Continuous variables were compared using Student’s t-test. We used the Kolmogorov–Smirnov test to confirm the normal distribution of continuous variables and they were all normally distributed. The chi-squared test or Fisher’s exact test was used to compare categorical variables. The Cox proportional hazards model was used to calculate the hazard ratios (HR) and 95% confidence intervals (CI). In the multivariable Cox proportional hazard model, age, sex, body mass index (BMI), income level, smoking status, alcohol consumption status, regular physical activity, baseline SBP (or DBP for ∆DBP analysis), diabetes mellitus, heart failure, cardiovascular disease, and prescription of antihypertensive drugs were adjusted. All tests were two-tailed, with p values ≤ 0.05 considered to indicate statistical significance. SAS version 9.2 (SAS Institute, Cary, NC, USA) was used for all the statistical analyses.

### Ethics approval

The current study was approved by the Institutional Review Board of Korea University Medicine Anam Hospital and the official review committee of the K-NHIS. Considering the retrospective nature of this study, the requirement for written informed consent was waived. The ethical guidelines of the 2013 Declaration of Helsinki and the legal medical regulations of the Republic of Korea were strictly undertaken throughout the study.

## Results

### Participants

**Figure 1:**
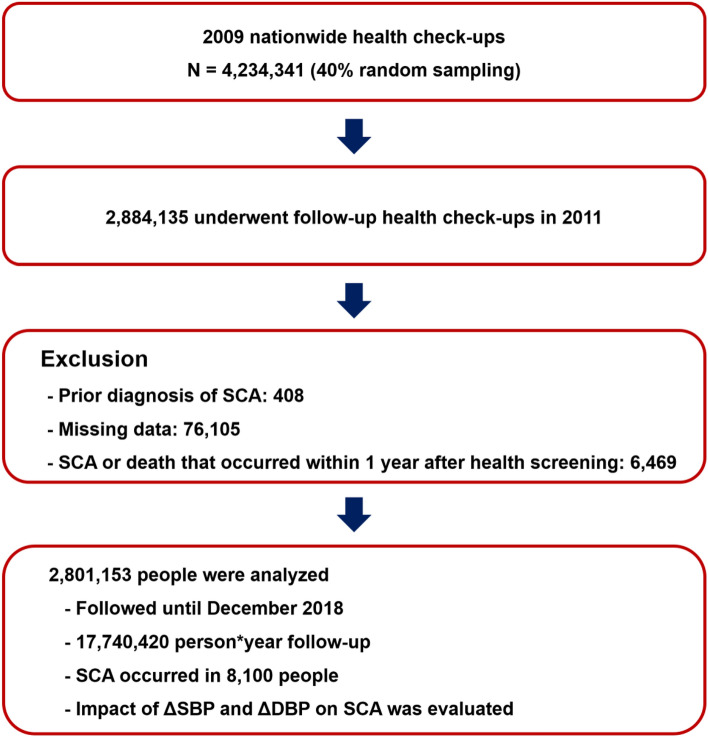
Study flow. *DM* diabetes mellitus, *HTN* hypertension, *ICD-10* International Classification of Disease, tenth edition, *SCA* sudden cardiac arrest.

We identified 10 million people aged > 20 years who underwent a nationwide health checkup in 2009, 40% of whom were randomly selected for this analysis (n = 4,234,341). Among the 4,234,341 people, 2,884,135 underwent follow-up health screening in 2011. Individuals with a prior diagnosis of SCA (n = 408), missing data (n = 76,105), and occurrence of death or SCA within 1 year after the 2011 health checkups (n = 6469) were excluded from the analysis. Since blood pressure was measured in both 2009 and 2011 health check-up, ∆SBP and ∆DBP were calculated and their impact on SCA risk was examined in this study. The flow of this study is summarized in Fig. 1. During 17,740,420 person-years of follow-up, 8100 SCA events were observed. The incidence per 1000 person*years was 0.457. Differences in the baseline characteristics of people who experienced SCA during follow-up are summarized in Table 1: they were older, likely to be male and current smokers, and had a higher prevalence of hypertension, diabetes mellitus, chronic kidney disease, and dyslipidemia^17^. People who had hypertension in 2009 were older, had higher BMI and waist circumference, and had a higher prevalence of diabetes mellitus, chronic kidney disease, and dyslipidemia (Table 2).

**Table 1:** Baseline demographics of participants with and without SCA.

	Sudden cardiac arrest		p-value
	No	Yes	
n	2,793,053	8100	
Age (year)	48.9 ± 13.5	63.1 ± 13.0	< 0.001
Male	1,595,232 (57.1%)	5952 (73.5%)	< 0.001
Body mass index (kg/m^2^)	23.8 ± 3.2	23.8 ± 3.5	0.369
Waist circumference (cm)	80.6 ± 9.0	83.5 ± 8.8	< 0.001
Smoking			< 0.001
Never-smoker	1,630,486 (58.4%)	3835 (47.4%)	
Ex-smoker	478,873 (17.2%)	1836 (22.7%)	
Current-smoker	683,694 (24.5%)	2429 (30.0%)	
Alcohol consumption			< 0.001
Non-drinker	1,413,877 (50.6%)	4728 (58.4%)	
Mild-drinker	1,176,540 (42.1%)	2712 (33.5%)	
Heavy-drinker	202,636 (7.3%)	660 (8.2%)	
Regular exercise	568,639 (20.4%)	1659 (20.5%)	0.785
Income (lowest 20%)	468,481 (16.8%)	1572 (19.4%)	< 0.001
Hypertension	781,544 (28.0%)	4708 (58.1%)	< 0.001
Systolic blood pressure (mmHg)	122.5 ± 14.6	128.8 ± 16.5	< 0.001
Diastolic blood pressure (mmHg)	76.4 ± 9.8	78.5 ± 10.7	< 0.001
Diabetes mellitus	257,011 (9.2%)	2113 (26.1%)	< 0.001
Glucose (mg/dL)	97.5 ± 22.3	108.6 ± 38.5	< 0.001
Dyslipidemia	572,161 (20.5%)	2460 (30.4%)	< 0.001
Cholesterol (mg/dL)	195.1 ± 36.4	192.3 ± 41.6	< 0.001
High-density lipoprotein (mg/dL)	55.2 ± 19.9	52.0 ± 18.0	< 0.001
Low-density lipoprotein (mg/dL)	114.6 ± 44.1	112.7 ± 48.5	< 0.001
Chronic kidney disease	145,927 (5.2%)	1194 (14.7%)	< 0.001
eGFR (mL/min/1.73 m^2^)	89.4 ± 37.3	82.1 ± 32.6	< 0.001

*eGFR* estimated glomerular filtration rate, *SCA* sudden cardiac arrest.

**Table 2:** Baseline demographics of participants with and without hypertension.

	Hypertension in 2009		p-value
	No	Yes	
	2,014,901	786,252	
Male	1,150,029 (57.1%)	451,155 (57.4%)	< 0.001
Age (year)	45.3 ± 12.3	58.0 ± 12.3	< 0.001
Age group			< 0.001
20–29	188,938 (9.4%)	7933 (1.0%)	
30–39	509,387 (25.3%)	53,231 (6.8%)	
40–49	584,809 (29.0%)	125,332 (15.9%)	
50–59	454,717 (22.6%)	225,815 (28.7%)	
60–69	194,226 (9.6%)	211,236 (26.9%)	
70–79	74,646 (3.7%)	141,730 (18.0%)	
80–	8,178 (0.4%)	20,975 (2.7%)	
Body mass index (kg/m^2^)	23.4 ± 3.0	25.0 ± 3.2	< 0.001
Waist circumference (cm)	79.2 ± 8.7	84.4 ± 8.5	< 0.001
Smoking			< 0.001
Never-smoker	1,162,688 (57.7%)	471,633 (60.0%)	
Ex-smoker	320,649 (15.9%)	160,060 (20.4%)	
Current-smoker	531,564 (26.4%)	154,559 (19.7%)	
Alcohol consumption			< 0.001
Non-drinker	979,572 (48.6%)	439,033 (55.8%)	
Mild-drinker	897,378 (44.5%)	281,874 (35.9%)	
Heavy-drinker	137,951 (6.9%)	65,345 (8.3%)	
Regular exercise	395,870 (19.7%)	174,428 (22.2%)	< 0.001
Income (lowest 20%)	323,581 (16.1%)	146,472 (18.6%)	< 0.001
Diabetes mellitus	103,078 (5.1%)	156,046 (19.9%)	< 0.001
Diabetes mellitus stage			< 0.001
Non-diabetic	1,499,523 (74.4%)	403,275 (51.3%)	
Impaired fasting glucose	412,300 (20.5%)	226,931 (28.9%)	
New onset diabetes	41,823 (2.1%)	33,462 (4.3%)	
Diabetic < 5 years	30,034 (1.5%)	53,219 (6.8%)	
Diabetic ≥ 5 years	31,221 (1.6%)	69,365 (8.8%)	
Glucose (mg/dL)	94.7 ± 19.2	104.7 ± 27.6	< 0.001
Systolic blood pressure (mmHg)	117.9 ± 11.3	134.4 ± 15.4	< 0.001
Diastolic blood pressure (mmHg)	73.7 ± 8.0	83.1 ± 10.8	< 0.001
Dyslipidemia	288,048 (14.3%)	286,573 (36.5%)	< 0.001
Dyslipidemia stage			< 0.001
Total cholesterol < 240 (mg/dL)	1,726,853 (85.7%)	499,679 (63.6%)	
Total cholesterol ≥ 240	164,478 (8.2%)	60,082 (7.6%)	
Total cholesterol ≥ 240 with medication	123,570 (6.1%)	226,491 (28.8%)	
Cholesterol (mg/dL)	194.7 ± 35.5	196.1 ± 38.6	< 0.001
High-density lipoprotein (mg/dL)	55.9 ± 19.0	53.2 ± 22.0	< 0.001
Low-density lipoprotein (mg/dL)	114.8 ± 43.5	114.0 ± 45.9	< 0.001
Chronic kidney disease	73,291 (3.6%)	73,830 (9.4%)	< 0.001
eGFR (mL/min/1.73m^2^)	90.9 ± 37.5	85.4 ± 36.3	< 0.001

*eGFR* estimated glomerular filtration rate, *SCA* sudden cardiac arrest.

### Temporal changes in blood pressure

Participants of this study were stratified into six groups according to ∆SBP (difference between 2009 and 2011) and baseline characteristics of each group are summarized in Table 3. Major differences were observed in sex, age, diabetes mellitus, and baseline blood pressure (measured in 2009). Although statistically significant differences were observed in low-density lipoprotein levels and eGFR, the degree of absolute difference was not clinically significant.

**Table 3:** Baseline demographics of participants stratified by ∆SBP.

	∆SBP						p-value
	ΔSBP < –20	− 20 ≤ ΔSBP < –10	− 10 ≤ ΔSBP < 10	10 ≤ ΔSBP < 20	20 ≤ ΔSBP < 40	40 ≤ ΔSBP	
	173,039	361,268	1,504,903	489,013	251,204	21,726	
Male	94,974 (54.9%)	205,138 (56.8%)	865,195 (57.5%)	283,037 (57.9%)	141,824 (56.5%)	11,016 (50.7%)	< 0.001
Age (year)	53.7 ± 13.9	49.4 ± 13.6	47.8 ± 13.2	48.5 ± 13.5	51.3 ± 14.1	58.6 ± 13.8	< 0.001
Age group							< 0.001
20–29	7436 (4.3%)	24,326 (6.7%)	113,978 (7.6%)	35,864 (7.3%)	14,811 (5.9%)	456 (2.1%)	
30–39	22,294 (12.9%)	69,334 (19.2%)	326,809 (21.7%)	100,691 (20.6%)	41,746 (16.6%)	1744 (8.0%)	
40–49	34,930 (20.2%)	89,452 (24.8%)	400,793 (26.6%)	125,315 (25.6%)	56,333 (22.4%)	3318 (15.3%)	
50–59	45,817 (26.4%)	89,935 (24.9%)	358,654 (23.8%)	118,236 (24.2%)	62,737 (25.0%)	5153 (23.7%)	
60–69	35,947 (20.8%)	55,315 (15.3%)	196,501 (13.1%)	68,436 (14.0%)	44,002 (17.5%)	5261 (24.2%)	
70–79	23,080 (13.3%)	29,094 (8.1%)	96,230 (6.4%)	35,722 (7.3%)	27,408 (10.9%)	4842 (22.3%)	
80–	3535 (2.0%)	3812 (1.1%)	11,938 (0.8%)	4749 (1.0%)	4167 (1.7%)	952 (4.4%)	
Body mass index (kg/m^2^)	24.1 ± 3.3	23.8 ± 3.2	23.7 ± 3.1	23.9 ± 3.2	24.2 ± 3.3	24.7 ± 3.5	< 0.001
Waist circumference (cm)	81.6 ± 9.2	80.5 ± 8.9	80.3 ± 8.9	80.8 ± 9.0	81.6 ± 9.0	83.4 ± 9.0	< 0.001
Smoking							< 0.001
Never-smoker	104,659 (60.5%)	212,072 (58.7%)	873,753 (58.1%)	281,801 (57.6%)	148,026 (58.9%)	14,010 (64.5%)	
Ex-smoker	29,632 (17.1%)	61,967 (17.2%)	258,112 (17.2%)	84,824 (17.4%)	42,715 (17.0%)	3459 (15.9%)	
Current-smoker	38,748 (22.4%)	87,229 (24.2%)	373,038 (24.8%)	122,388 (25.0%)	60,463 (24.1%)	4257 (19.6%)	
Alcohol consumption							< 0.001
Non-drinker	96,084 (55.5%)	186,968 (51.8%)	749,853 (49.8%)	242,850 (49.7%)	130,123 (51.8%)	12,727 (58.6%)	
Mild-drinker	64,456 (37.3%)	149,544 (41.4%)	647,876 (43.1%)	209,216 (42.8%)	101,031 (40.2%)	7129 (32.8%)	
Heavy-drinker	12,499 (7.2%)	24,756 (6.9%)	107,174 (7.1%)	36,947 (7.6%)	20,050 (8.0%)	1870 (8.6%)	
Regular Exercise	35,783 (20.7%)	74,886 (20.7%)	306,969 (20.4%)	98,775 (20.2%)	49,898 (19.9%)	3987 (18.4%)	< 0.001
Income (lowest 20%)	32,176 (18.6%)	61,456 (17.0%)	245,069 (16.3%)	82,469 (16.9%)	44,617 (17.8%)	4266 (19.6%)	< 0.001
Diabetes mellitus	23,955 (13.8%)	35,059 (9.7%)	123,639 (8.2%)	43,924 (9.0%)	28,677 (11.4%)	3870 (17.8%)	< 0.001
Diabetes mellitus stage							< 0.001
Non-diabetic	107,124 (61.9%)	246,992 (68.4%)	1,047,958 (69.6%)	330,204 (67.5%)	158,913 (63.3%)	11,607 (53.4%)	
Impaired fasting glucose	41,960 (24.3%)	79,217 (21.9%)	333,306 (22.2%)	114,885 (23.5%)	63,614 (25.3%)	6249 (28.8%)	
New onset diabetes	5736 (3.3%)	9358 (2.6%)	36,992 (2.5%)	13,487 (2.8%)	8640 (3.4%)	1072 (4.9%)	
Diabetic < 5 years	8527 (4.9%)	11,783 (3.3%)	39,581 (2.6%)	13,579 (2.8%)	8642 (3.4%)	1141 (5.3%)	
Diabetic ≥ 5 years	9692 (5.6%)	13,918 (3.9%)	47,066 (3.1%)	16,858 (3.5%)	11,395 (4.5%)	1657 (7.6%)	
Glucose (mg/dL)	100.2 ± 25.7	97.4 ± 22.9	96.7 ± 21.4	97.6 ± 22.0	99.6 ± 24.2	104.1 ± 28.9	< 0.001
Systolic blood pressure in 2011 (mmHg)	113.4 ± 14.0	114.7 ± 12.6	120.6 ± 12.5	128.2 ± 12.3	137.1 ± 14.1	160.4 ± 17.3	< 0.001
Systolic blood pressure in 2009 (mmHg)	143.46 ± 16.72	130.63 ± 12.59	121.88 ± 12.32	115.5 ± 12.07	112.64 ± 13.31	114.4 ± 14.62	< 0.001
Diastolic blood pressure in 2011 (mmHg)	72.1 ± 9.6	72.7 ± 9.1	75.4 ± 9.0	79.1 ± 9.1	83.3 ± 10.1	93.4 ± 12.6	< 0.001
Diastolic blood pressure in 2009 (mmHg)	86.15 ± 11.61	80.06 ± 9.46	76.08 ± 9.05	73.37 ± 8.98	72.03 ± 9.29	73.23 ± 9.92	< 0.001
Hypertension with medication (in 2011)	71,782 (41.5%)	84,190 (23.3%)	257, 886 (17.1%)	91,272 (18.7%)	67,213 (26.8%)	11,127 (51.2%)	< 0.001
Number of antihypertensive medications	0.78 ± 0.11	0.42 ± 0.87	0.3 ± 0.75	0.32 ± 0.78	0.47 ± 0.91	0.97 ± 1.17	< 0.001
Dyslipidemia	46,450 (26.8%)	76,278 (21.1%)	287,961 (19.1%)	98,271 (20.1%)	58,836 (23.4%)	6825 (31.4%)	< 0.001
Dyslipidemia stage							< 0.001
Total cholesterol < 240 (mg/dL)	126,589 (73.2%)	284,990 (78.9%)	1,216,942 (80.9%)	390,742 (79.9%)	192,368 (76.6%)	14,901 (68.6%)	
Total cholesterol ≥ 240	12,784 (7.4%)	27,099 (7.5%)	118,514 (7.9%)	41,023 (8.4%)	22,855 (9.1%)	2285 (10.5%)	
Total cholesterol ≥ 240 with medication	33,666 (19.5%)	49,179 (13.6%)	169,447 (11.3%)	57,248 (11.7%)	35,981 (14.3%)	4540 (20.9%)	
Cholesterol (mg/dL)	193.8 ± 37.2	193.9 ± 36.2	194.6 ± 36.0	195.9 ± 36.4	198.0 ± 38.0	201.6 ± 39.1	< 0.001
High-density lipoprotein (mg/dL)	54.1 ± 19.5	54.9 ± 18.5	55.3 ± 19.9	55.3 ± 18.4	55.2 ± 24.9	54.7 ± 17.8	< 0.001
Low-density lipoprotein (mg/dL)	113.8 ± 42.3	114.2 ± 41.4	114.5 ± 44.5	114.9 ± 44.2	115.6 ± 47.4	116.6 ± 39.8	< 0.001
Chronic kidney disease	13,138 (7.6%)	19,632 (5.4%)	72,631 (4.8%)	24,277 (5.0%)	15,296 (6.1%)	2147 (9.9%)	< 0.001
eGFR (mL/min/1.73 m^2^)	87.5 ± 36.2	89.1 ± 36.4	89.8 ± 37.2	89.6 ± 37.8	88.6 ± 38.0	86.3 ± 39.8	< 0.001
Heart failure	363 (0.21%)	397 (0.11%)	1203 (0.08%)	391 (0.08%)	326 (0.13%)	69 (0.32%)	< 0.001
Cardiovascular disease	6471 (3.7%)	8056 (2.2%)	26,335 (1.8%)	9291 (1.9%)	6832 (2.7%)	1142 (5.3%)	< 0.001

*eGFR* estimated glomerular filtration rate, *SBP* systolic blood pressure.

In univariate analysis, ∆SBP showed a significant association with the risk of SCA (Supplementary Table S1) with people having extreme changes being associated with the highest risk (∆SBP within 10 mmHg as a reference group): HR 2.07 (95% CI 1.92–2.23; p < 0.001) and 3.38 (95% CI 2.90–3.93; p < 0.001) for ∆SBP < –20 and ≥ 40 group, respectively. In the multivariate model, baseline SBP in 2009 was adjusted for, in addition to other covariates, as baseline SBP showed the most prominent difference across the six groups. Compared with the reference group, people with ∆SBP < − 20 (adjusted HR 1.06; 95% CI 0.98–1.15) and − 20 ≤ ∆SBP < − 10 (adjusted HR 0.98; 95% CI 0.91–1.05) demonstrated no significant difference regarding SCA risk. However, positive ∆SBP (10 ≤ ∆SBP < 20) was associated with a significantly increased risk of SCA (adjusted HR 1.11; 95% CI 1.04–1.19; p = 0.001). The degree of increased SCA risk showed a linear association with ∆SBP with 20 ≤ ∆SBP < 40 group (adjusted HR 1.40; 95% CI 1.30–1.50; p < 0.001) and ∆SBP ≥ 40 group (adjusted HR 1.88; 95% CI 1.62–2.19; p < 0.001) having greater risk of SCA. Further adjustment for heart failure, cardiovascular disease, and prescription of antihypertensive medications showed similar results with ∆SBP ≥ 40 group having 67.4% increased risk of SCA (adjusted HR 1.67; 95% CI 1.44–1.95; p < 0.001; Supplementary Table S1).

The risk of SCA was also associated with temporal changes in DBP. Compared with the reference group (− 5 ≤ ΔDBP < 5), people with increased ∆DBP showed significantly higher risk of SCA: (i) 5 ≤ ΔDBP < 15 (adjusted HR 1.10; 95% CI 1.04–1.17; p = 0.001; Table 4); (ii) 15 ≤ ΔDBP < 25 (adjusted HR 1.28; 95% CI 1.17–1.40; p < 0.001; Table 4); (iii) ∆DBP ≥ 25 group (adjusted HR 1.61; 95% CI 1.37–1.89; p < 0.001; Table 4). In ∆DBP ≥ 25 group, the risk of SCA was 51.6% higher after further adjusting for heart failure, cardiovascular disease, and prescription of antihypertensive medications (adjusted HR 1.52; 95% CI 1.33–1.73; p < 0.001; Supplementary Table S1).

**Table 4:**
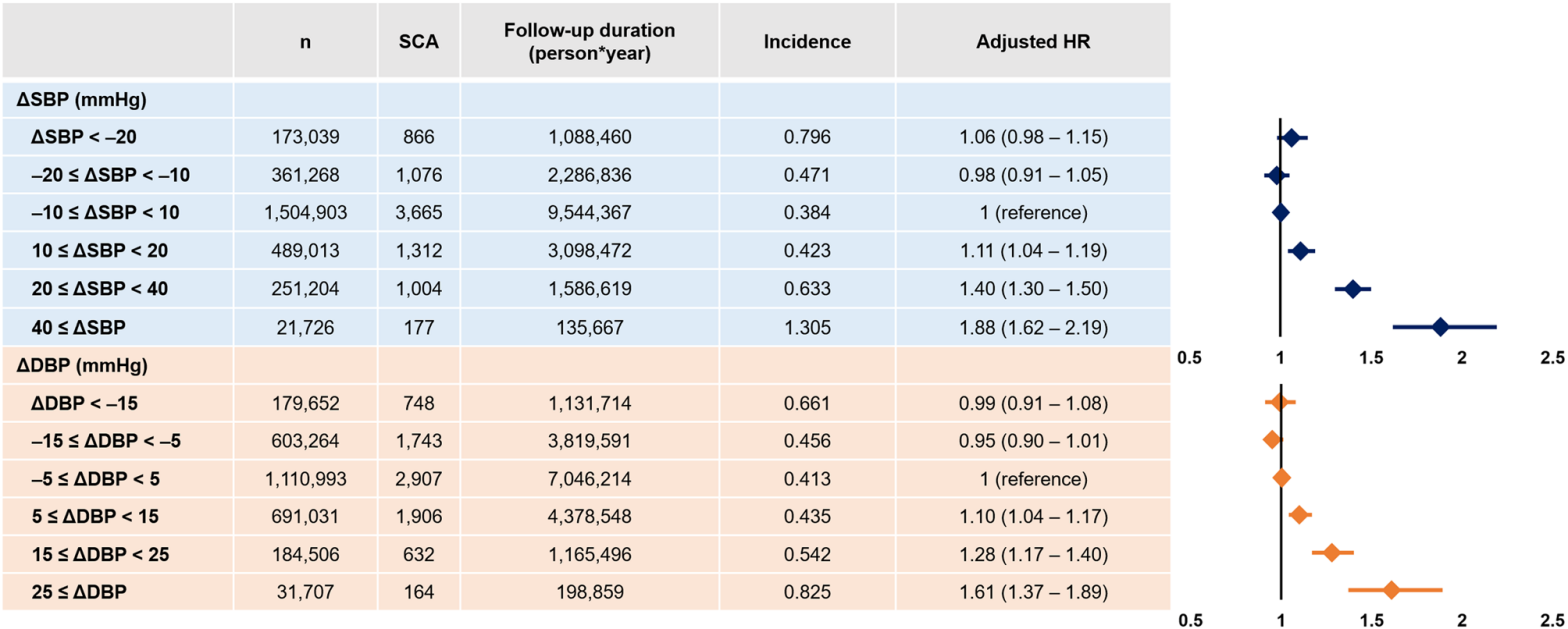
The risk of SCA according to ΔSBP and ΔDBP.

Incidence is per 1000 person*year follow-up.

*DBP* diastolic blood pressure, *SBP* systolic blood pressure, *SCA* sudden cardiac arrest.

Hazard ratios (HRs) were adjusted for age, sex, body mass index, smoking status, alcohol consumption, regular physical activity, income level, and baseline blood pressure (measured in 2009).

### Subgroup analysis

**Figure 2:**
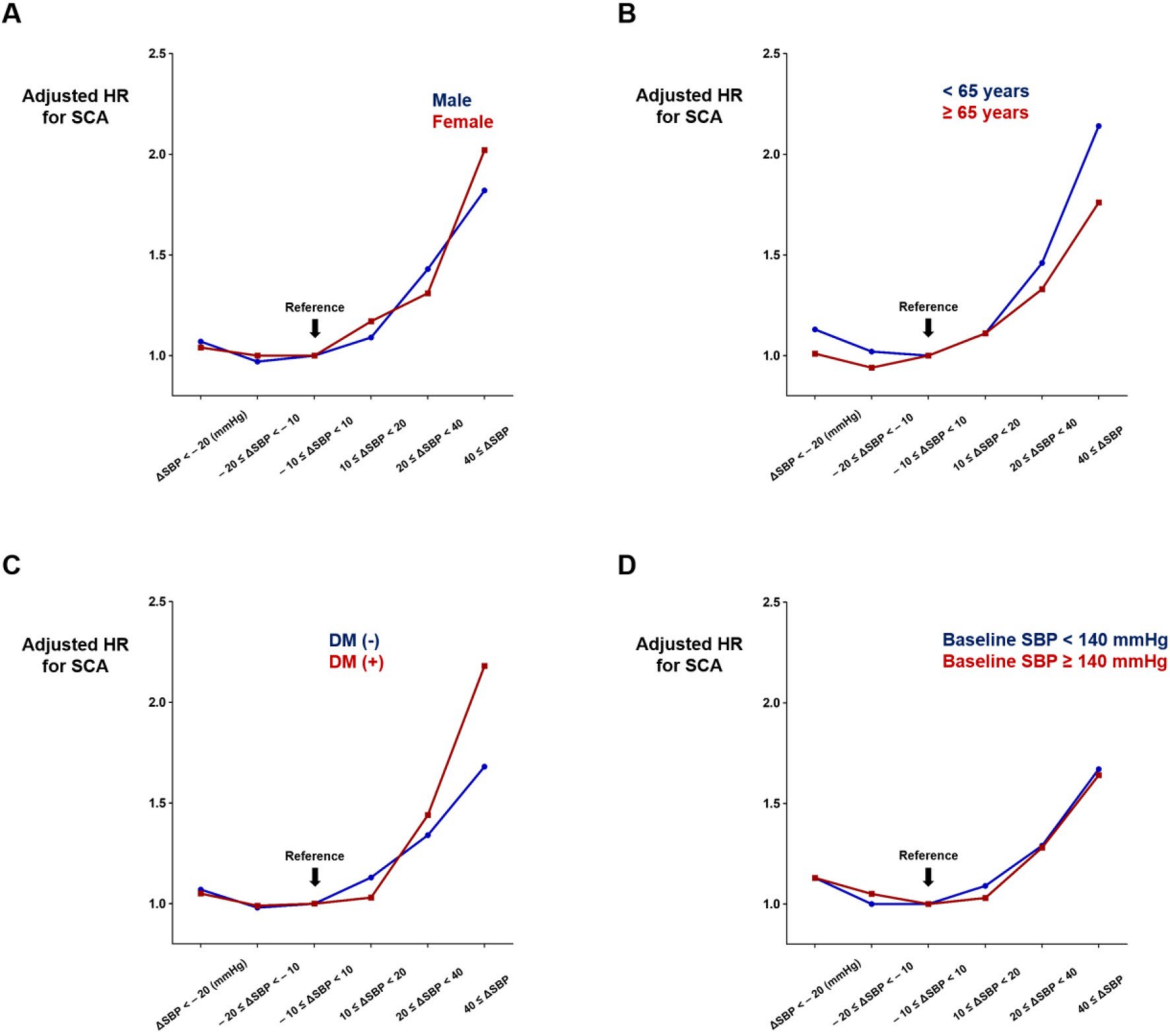
Impact of ∆SBP on SCA in various subgroups. The association between ∆SBP and SCA risk was not affected by sex category (**A**), age (**B**), diabetes mellitus (**C**), and baseline SBP (**D**). Hazard ratios (HRs) were adjusted for age, sex, body mass index, smoking status, alcohol consumption, regular physical activity, income level, and baseline blood pressure (measured in 2009). *DM* diabetes mellitus, *SBP* systolic blood pressure, *SCA* sudden cardiac arrest.

The association between ∆SBP and SCA was evaluated according to age, sex category, presence of diabetes mellitus, and baseline SBP (measured in 2009), and no significant interactions were observed (Fig. 2 and Supplementary Table S2). Adjusted HR was numerically higher in people with diabetes mellitus and ∆SBP ≥ 40 as compared with non-diabetes and ∆SBP ≥ 40 (adjusted HR 2.18 vs. 1.68) but the p-value for interaction was insignificant (p = 0.102). In people with baseline SBP < 140, ∆SBP < 20 was associated with 12.7% increased risk of SCA compared with − 10 ≤ ∆SBP < 10 group (adjusted HR 1.13; 95% CI 1.03–1.24; p = 0.011; Fig. 2 and Supplementary Table S2).

## Discussion

This study revealed that (i) a temporal increase in blood pressure was significantly associated with the occurrence of SCA; (ii) the association was consistent for both SBP and DBP; (iii) the greater the increase in blood pressure, the higher the risk of SCA regardless of the patient subgroup; and (iv) a temporal decrease in SBP or DBP did not show a protective effect against SCA. Despite the challenges of low incidence and the need for long-term follow-up, we were able to analyze the association between temporal changes in blood pressure and SCA risk using nationwide healthcare insurance data. The strengths of this study are the sequential measurement of blood pressure in a large population-based cohort and the long-term clinical follow-up.

### Blood pressure and SCA

Our previous study demonstrated that hypertension and prehypertension were significantly associated with an increased risk of SCA^10,17^. This study revealed that temporal elevation in blood pressure is also associated with an increased risk of SCA. In this study, HRs were adjusted for baseline blood pressure, and temporal elevation of blood pressure was associated with SCA risk in both people with SBP < 140 and ≥ 140 suggesting not only baseline blood pressure but also temporal elevation in blood pressure can contribute to SCA risk (Fig. 2 and Supplementary Table S2). The association between ∆SBP and the risk of SCA was not affected by age, sex, presence of diabetes mellitus, and baseline SBP, and other confounders such as BMI, smoking status, alcohol consumption, regular physical activity, and income level, were adjusted for in the multivariate model. Our study indicates that a temporal elevation in blood pressure can be associated with an increased risk of SCA.

### Temporal decrease in blood pressure

Preventing elevated blood pressure may contribute to the primary prevention of SCA, according to the current analysis. However, a temporal decrease in blood pressure was not associated with a decreased risk of SCA in our study. The degree of decrease in both SBP and DBP also showed no association with the risk of SCA. However, the mechanisms underlying this lack of association are not fully understood. There may be an irreversible portion of target organ damage during periods of hypertension, emphasizing the importance of the early detection of hypertension and continuous, not temporary, blood pressure control. Our study is in accordance with the ACCORD blood pressure trial, which found no benefit of targeting SBP less than 120 mmHg compared to < 140 mmHg in patients with type 2 diabetes mellitus^18^. Maintaining optimal blood pressure throughout the clinical course can be more beneficial than aggressively decreasing blood pressure within a short period of time.

However, the optimal level of blood pressure control remains controversial. The 2017 ACC/AHA/AAPA/ABC/ACPM/AGS/APhA/ASH/ASPC/NMA/PCNA hypertension guidelines presented a strict standard for blood pressure control with a target of 130/80 mmHg compared with 140/90 mmHg in the previous guidelines and the 2018 European guideline^19–21^. In terms of SCA risk, our study revealed no benefit of blood pressure reduction compared with blood pressure maintenance. Furthermore, ∆SBP < − 20 in people with baseline SBP < 140 showed a statistically significant increase in the risk of SCA (adjusted HR 1.13, 95% CI 1.03–1.24; Supplementary Table S2). In contrast to this study, the SPRINT trial demonstrated that in patients at high risk of cardiovascular events, targeting SBP less than 120 mmHg as compared with SBP < 140 mmHg resulted in lower rates of fatal and nonfatal major cardiovascular events and death from any cause^22^. Identification the ideal degree of blood pressure control and specific subgroups to prevent SCA and other cardiovascular complications remain to be elucidated in future studies.

### Limitations

This study had several limitations. First, owing to the retrospective nature and utilization of nationwide healthcare insurance data, coding inaccuracies may exist. However, our coding strategy for various medical conditions has been validated in previous publications^14–16,23^. Second, our analysis focused on the occurrence of SCA; the clinical course of SCA events was not available, and the type of treatment performed for SCA events could not be analyzed. Third, the current study was exclusively based on East Asians, and the extrapolation of our results to different ethnic groups requires caution. Fourth, our results suggest an association between the temporal elevation of blood pressure and SCA risk. Whether medical treatments for hypertension will reduce the risk of SCA requires further evaluation. Our sequential blood pressure measurements were separated by 2 years. Long-term changes in blood pressure can have different influences on the risk of SCA. Multiple BP measurements (for example, more than twice) can also have additional clinical implications. Fifth, the blood pressure measurement process was not standardized, because the measurements were performed in multiple primary care centers and hospitals across the nation.

## Conclusions

A temporal increase in blood pressure was significantly associated with the occurrence of SCA, which was consistent among various subgroups. In contrast, a temporal decrease in blood pressure failed to demonstrate any significant association with a reduced risk of SCA.

### Supplementary Information

Supplementary Tables.

## Data Availability

All data generated or analyzed during this study are included in this article. Further inquiries can be directed to the corresponding authors. Raw data cannot be shared due to the legal regulations of the Republic of Korea and the policy of the K-NHIS.

## Abbreviations

BMI: Body mass index
CI: Confidence interval
DBP: Diastolic blood pressure
FBG: Fasting blood glucose
HR: Hazard ratio
ICD: International classification of disease
K-NHIS: Korean National Health Insurance Service
SBP: Systolic blood pressure
SCA: Sudden cardiac arrest

## References

[1] Kragholm K (2017). Bystander efforts and 1-year outcomes in out-of-hospital cardiac arrest. N. Engl. J. Med..

[2] Brady WJ, Mattu A, Slovis CM (2019). Lay responder care for an adult with out-of-hospital cardiac arrest. N. Engl. J. Med..

[3] Ricceri S (2021). Factors predisposing to survival after resuscitation for sudden cardiac arrest. J. Am. Coll. Cardiol..

[4] Desch S (2021). Angiography after out-of-hospital cardiac arrest without ST-segment elevation. N. Engl. J. Med..

[5] Dankiewicz J (2021). Hypothermia versus Normothermia after Out-of-Hospital Cardiac Arrest. N. Engl. J. Med..

[6] Kudenchuk PJ (2016). Amiodarone, lidocaine, or placebo in out-of-hospital cardiac arrest. N. Engl. J. Med..

[7] Cummins RO, Ornato JP, Thies WH, Pepe PE (1991). Improving survival from sudden cardiac arrest: The "chain of survival" concept. A statement for health professionals from the Advanced Cardiac Life Support Subcommittee and the Emergency Cardiac Care Committee, American Heart Association. Circulation.

[8] Ong MEH, Perkins GD, Cariou A (2018). Out-of-hospital cardiac arrest: Prehospital management. Lancet.

[9] Stiell IG (1999). Modifiable factors associated with improved cardiac arrest survival in a multicenter basic life support/defibrillation system: OPALS study phase I results. Ann. Emerg. Med..

[10] Kim YG (2022). Hypertension and diabetes including their earlier stage are associated with increased risk of sudden cardiac arrest. Sci. Rep..

[11] Flint AC (2019). Effect of systolic and diastolic blood pressure on cardiovascular outcomes. N. Engl. J. Med..

[12] Itoga NK, Tawfik DS, Montez-Rath ME, Chang TI (2021). Contributions of systolic and diastolic blood pressures to cardiovascular outcomes in the ALLHAT study. J. Am. Coll. Cardiol..

[13] Ma Y (2020). Blood pressure variation and subclinical brain disease. J. Am. Coll. Cardiol..

[14] Kim YG (2020). Non-genetic risk factors for atrial fibrillation are equally important in both young and old age: A nationwide population-based study. Eur. J. Prev. Cardiol..

[15] Kim YG (2019). The impact of body weight and diabetes on new-onset atrial fibrillation: A nationwide population based study. Cardiovasc. Diabetol..

[16] Kim YG (2019). Impact of the duration and degree of hypertension and body weight on new-onset atrial fibrillation: A nationwide population-based study. Hypertension.

[17] Kim YG (2022). Metabolic syndrome, gamma-glutamyl transferase, and risk of sudden cardiac death. J. Clin. Med..

[18] Group AS (2010). Effects of intensive blood-pressure control in type 2 diabetes mellitus. N. Engl. J. Med..

[19] Whelton PK (2018). A guideline for the prevention, detection, evaluation and management of high blood pressure. A report of the American College of Cardiology/American Heart Association task force on clinical practice guidelines. Hypertension.

[20] Williams B (2018). 2018 ESC/ESH Guidelines for the management of arterial hypertension: The Task Force for the management of arterial hypertension of the European Society of Cardiology (ESC) and the European Society of Hypertension (ESH). Eur. Heart J..

[21] James P, Oparil S, Carter B (2014). 20I4 evidence-based guideline for the management of high blood pressure in adults: Report from the panel members appointed to the Eight Joint National Committee (JNC 8) [published correction appears in JAMA. 20I4; 3II (I7): I809]. JAMA.

[22] Group SR (2015). A randomized trial of intensive versus standard blood-pressure control. N. Engl. J. Med..

[23] Kim YG (2021). Different influence of blood pressure on new-onset atrial fibrillation in pre- and postmenopausal women: A nationwide population-based study. Hypertension.

