# Supplementary material for: Temporal elevation of blood pressure is associated with increased risk of sudden cardiac arrest
Source: Sci Rep. 2024 Jan 27;14:2289. doi: 10.1038/s41598-024-52859-x (PMC10821940; doi:10.1038/s41598-024-52859-x)
Supplement: Supplementary file 1 — Supplementary Tables. [file 41598_2024_52859_MOESM1_ESM.pdf]

Temporal Elevation of Blood Pressure is Associated with Increased Risk of Sudden Cardiac Arrest

Yun Gi Kim,[1#] Kyongjin Min,[2#] Joo Hee Jeong,[1] Seung-Young Roh,[3] Kyung-Do Han,[4] Jaemin Shim,[1] Jong-Il Choi,[1*] and Young-Hoon Kim[1]

[1]Division of Cardiology, Department of Internal Medicine, Korea University College of Medicine and Korea University Anam Hospital, Seoul, Republic of Korea

[2]Division of Cardiology, Department of Internal Medicine, Sanggye Paik Hospital, Inje University College of Medicine, Seoul, Republic of Korea

[3]Division of Cardiology, Department of Internal Medicine, Korea University College of Medicine and Korea University Guro Hospital, Seoul, Republic of Korea

[4]Department of Statistics and Actuarial Science, Soongsil University, Seoul, Republic of Korea

*Address for correspondence: Jong-Il Choi, MD, PhD, MHSc

[1]Division of Cardiology, Department of Internal Medicine, Korea University College of Medicine and Korea University Anam Hospital, Seoul, Republic of Korea

73 Goryeodae-ro, Seongbuk-gu, Seoul 02841, Republic of Korea

Tel: 82-2-920-5445

Fax: 82-2-927-1478

E-mail: jongilchoi@korea.ac.kr

# The first two authors contributed equally to this work.

Running title: Blood pressure control and sudden cardiac arrest

Disclosure: The authors have nothing to disclose.

Total word count: 6,659

Supplementary Table S1. The risk of SCA according to ΔSBP and ΔDBP (including unadjusted models).

	n	SCA	Follow-up duration (person*year)	Incidence	Hazard ratio with 95% confidence interval			
					Univariate	Multivariate model 1	Multivariate model 2	Multivariate model 3
**ΔSBP (mmHg)**								
ΔSBP < –20	173,039	866	1,088,460	0.796	2.07 (1.92 – 2.23)	1.36 (1.26 – 1.47)	1.06 (0.98 – 1.15)	1.00 (0.92 – 1.08)
–20 ≤ ΔSBP < –10	361,268	1,076	2,286,836	0.471	1.23 (1.15 – 1.31)	1.08 (1.01 – 1.16)	0.98 (0.91 – 1.05)	0.95 (0.89 – 1.02)
–10 ≤ ΔSBP < 10	1,504,903	3,665	9,544,367	0.384	1 (reference)	1 (reference)	1 (reference)	1 (reference)
10 ≤ ΔSBP < 20	489,013	1,312	3,098,472	0.423	1.10 (1.04 – 1.17)	1.03 (0.97 – 1.10)	1.11 (1.04 – 1.19)	1.08 (1.01 – 1.15)
20 ≤ ΔSBP < 40	251,204	1,004	1,586,619	0.633	1.65 (1.53 – 1.76)	1.25 (1.16 – 1.34)	1.40 (1.30 – 1.50)	1.33 (1.24 – 1.43)
40 ≤ ΔSBP	21,726	177	135,667	1.305	3.38 (2.90 – 3.93)	1.66 (1.43 – 1.93)	1.88 (1.62 – 2.19)	1.67 (1.44 – 1.95)
**ΔDBP (mmHg)**								
ΔDBP < –15	179,652	748	1,131,714	0.661	1.60 (1.48 – 1.74)	1.19 (1.10 – 1.29)	0.99 (0.91 – 1.08)	0.98 (0.93 – 1.04)
–15 ≤ ΔDBP < –5	603,264	1,743	3,819,591	0.456	1.11 (1.04 – 1.17)	1.02 (0.96 – 1.08)	0.95 (0.90 – 1.01)	0.94 (0.89 – 1.00)
–5 ≤ ΔDBP < 5	1,110,993	2,907	7,046,214	0.413	1 (reference)	1 (reference)	1 (reference)	1 (reference)
5 ≤ ΔDBP < 15	691,031	1,906	4,378,548	0.435	1.06 (1.00 – 1.12)	1.04 (0.98 – 1.10)	1.10 (1.04 – 1.17)	1.08 (1.03 – 1.14)
15 ≤ ΔDBP < 25	184,506	632	1,165,496	0.542	1.31 (1.21 – 1.43)	1.16 (1.06 – 1.26)	1.28 (1.17 – 1.40)	1.22 (1.13 – 1.32)
25 ≤ ΔDBP	31,707	164	198,859	0.825	2.00 (1.71 – 2.34)	1.42 (1.21 – 1.66)	1.61 (1.37 – 1.89)	1.52 (1.33 – 1.73)

Incidence is per 1,000 person*year follow-up.

DBP: diastolic blood pressure; SBP: systolic blood pressure; SCA: sudden cardiac arrest.

Multivariate model 1: adjusted for age and sex.

Multivariate model 2: adjusted for age, sex, body mass index, smoking status, alcohol consumption, regular physical activity, income level,

baseline blood pressure (measured in 2009).

Multivariate model 3: adjusted for age, sex, body mass index, smoking status, alcohol consumption, regular physical activity, income level, baseline blood pressure (measured in 2009), diabetes mellitus, heart failure, cardiovascular disease, and antihypertensive medication.

Supplementary Table S2. The risk of SCA according to ΔSBP in various subgroups.

	n	SCA	Follow-up duration (person*year)	Incidence	Hazard ratio with 95% confidence interval	
					Univariate	Multivariate model
**Male**						
ΔSBP < –20	94,974	625	592,198	1.06	2.11 (1.94 – 2.30)	1.07 (0.97 – 1.17)
–20 ≤ ΔSBP < –10	205,138	788	1,292,385	0.61	1.22 (1.13 – 1.32)	0.97 (0.90 – 1.05)
–10 ≤ ΔSBP < 10	865,195	2,734	5,465,577	0.50	1 (reference)	1 (reference)
10 ≤ ΔSBP < 20	283,037	953	1,785,276	0.53	1.07 (0.99 – 1.15)	1.09 (1.01 – 1.18)
20 ≤ ΔSBP < 40	141,824	738	889,600	0.83	1.66 (1.53 – 1.80)	1.43 (1.31 – 1.55)
40 ≤ ΔSBP	11,016	114	67,724	1.68	3.36 (2.78 – 4.05)	1.82 (1.51 – 2.20)
**Female**						
ΔSBP < –20	78,065	241	496,262	0.49	2.12 (1.84 – 2.44)	1.04 (0.90 – 1.20)
–20 ≤ ΔSBP < –10	156,130	288	994,451	0.29	1.27 (1.11 – 1.45)	1.00 (0.88 – 1.15)
–10 ≤ ΔSBP < 10	639,708	931	4,078,790	0.23	1 (reference)	1 (reference)
10 ≤ ΔSBP < 20	205,976	359	1,313,196	0.27	1.20 (1.06 – 1.35)	1.17 (1.04 – 1.33)
20 ≤ ΔSBP < 40	109,380	266	697,019	0.38	1.67 (1.45 – 1.91)	1.31 (1.14 – 1.50)
40 ≤ ΔSBP	10,710	63	67,943	0.93	4.01 (3.11 – 5.18)	2.02 (1.56 – 2.60)
**Age < 65 years**						
ΔSBP < –20	131,949	340	837,018	0.41	1.78 (1.59 – 2.00)	1.13 (1.00 – 1.27)
–20 ≤ ΔSBP < –10	307,786	534	1,956,609	0.27	1.20 (1.09 – 1.32)	1.02 (0.93 – 1.12)
–10 ≤ ΔSBP < 10	1,326,559	1,926	8,437,479	0.23	1 (reference)	1 (reference)
10 ≤ ΔSBP < 20	423,575	652	2,692,899	0.24	1.06 (0.97 – 1.16)	1.11 (1.02 – 1.21)
20 ≤ ΔSBP < 40	202,859	438	1,287,946	0.34	1.49 (1.34 – 1.65)	1.46 (1.32 – 1.62)
40 ≤ ΔSBP	13,710	53	86,582	0.61	2.68 (2.04 – 3.52)	2.14 (1.63 – 2.81)
**Age ≥ 65 years**						

ΔSBP < –20	41,090	526	251,442	2.09	1.33 (1.21 – 1.47)	1.01 (0.91 – 1.13)
–20 ≤ ΔSBP < –10	53,482	542	330,227	1.64	1.05 (0.95 – 1.15)	0.94 (0.85 – 1.04)
–10 ≤ ΔSBP < 10	178,344	1,739	1,106,887	1.57	1 (reference)	1 (reference)
10 ≤ ΔSBP < 20	65,438	660	405,573	1.63	1.04 (0.95 – 1.13)	1.11 (1.01 – 1.22)
20 ≤ ΔSBP < 40	48,345	566	298,673	1.90	1.21 (1.10 – 1.33)	1.33 (1.21 – 1.47)
40 ≤ ΔSBP	8,016	124	49,085	2.53	1.61 (1.34 – 1.93)	1.76 (1.47 – 2.12)
**DM (-)**						
ΔSBP < –20	149,084	601	940,727	0.64	2.02 (1.85 – 2.20)	1.07 (0.97 – 1.17)
–20 ≤ ΔSBP < –10	326,209	786	2,068,276	0.38	1.20 (1.11 – 1.30)	0.98 (0.90 – 1.06)
–10 ≤ ΔSBP < 10	1,381,264	2,777	8,771,659	0.32	1 (reference)	1 (reference)
10 ≤ ΔSBP < 20	445,089	1,003	2,824,456	0.36	1.12 (1.04 – 1.21)	1.13 (1.05 – 1.21)
20 ≤ ΔSBP < 40	222,527	709	1,408,716	0.50	1.59 (1.46 – 1.72)	1.34 (1.24 – 1.46)
40 ≤ ΔSBP	17,856	111	112,067	0.99	3.11 (2.57 – 3.76)	1.68 (1.38 – 2.03)
**DM (+)**						
ΔSBP < –20	23,955	265	147,733	1.79	1.56 (1.36 – 1.79)	1.05 (0.91 – 1.21)
–20 ≤ ΔSBP < –10	35,059	290	218,560	1.33	1.16 (1.01 – 1.32)	0.99 (0.87 – 1.14)
–10 ≤ ΔSBP < 10	123,639	888	772,708	1.15	1 (reference)	1 (reference)
10 ≤ ΔSBP < 20	43,924	309	274,016	1.13	0.98 (0.86 – 1.12)	1.03 (0.91 – 1.17)
20 ≤ ΔSBP < 40	28,677	295	177,903	1.66	1.44 (1.26 – 1.64)	1.44 (1.26 – 1.64)
40 ≤ ΔSBP	3,870	66	23,600	2.80	2.43 (1.89 – 3.12)	2.18 (1.70 – 2.80)
**Baseline SBP < 140 mmHg**						
ΔSBP < –20	166,405	798	1,047,414	0.76	2.18 (2.02 – 2.36)	1.13 (1.03 – 1.24)
–20 ≤ ΔSBP < –10	348,554	974	2,207,463	0.44	1.27 (1.18 – 1.36)	1.00 (0.93 – 1.08)
–10 ≤ ΔSBP < 10	1,411,657	3,122	8,959,709	0.35	1 (reference)	1 (reference)
10 ≤ ΔSBP < 20	405,418	879	2,573,325	0.34	0.98 (0.91 – 1.06)	1.09 (1.01 – 1.18)
20 ≤ ΔSBP < 40	151,285	386	959,505	0.40	1.15 (1.04 – 1.28)	1.29 (1.15 – 1.44)

40 ≤ ΔSBP	2,107	7	13,285	0.53	1.51 (0.72 – 3.17)	1.67 (0.79 – 3.51)
**Baseline SBP ≥ 140 mmHg**						
ΔSBP < –20	6,634	68	41,046	1.66	1.79 (1.39 – 2.30)	1.13 (0.87 – 1.46)
–20 ≤ ΔSBP < –10	12,714	102	79,373	1.29	1.38 (1.12 – 1.71)	1.05 (0.84 – 1.30)
–10 ≤ ΔSBP < 10	93,246	543	584,658	0.93	1 (reference)	1 (reference)
10 ≤ ΔSBP < 20	83,595	433	525,148	0.82	0.89 (0.78 – 1.01)	1.03 (0.91 – 1.17)
20 ≤ ΔSBP < 40	99,919	618	627,114	0.99	1.06 (0.94 – 1.19)	1.28 (1.13 – 1.45)
40 ≤ ΔSBP	19,619	170	122,382	1.39	1.49 (1.25 – 1.77)	1.64 (1.37 – 1.97)

Incidence is per 1,000 person*year follow-up.

DM: diabetes mellitus; SBP: systolic blood pressure; SCA: sudden cardiac arrest.

Multivariate model: adjusted for age, sex, body mass index, smoking status, alcohol consumption, regular physical activity, income level, baseline blood pressure (measured in 2009).
